# The Effects of Human Visual Sensory Stimuli on N1b Amplitude: An EEG Study

**DOI:** 10.3390/jcm9092837

**Published:** 2020-09-02

**Authors:** Damien Moore, Toshikazu Ikuta, Paul D. Loprinzi

**Affiliations:** 1 Exercise & Memory Laboratory, Department of Health, Exercise Science and Recreation Management, The University of Mississippi, University, MS 38677, USA; dcmoore3@go.olemiss.edu; 2 Digital Neuroscience Laboratory, Department of Communication Sciences and Disorders, The University of Mississippi, University, MS 38677, USA; tikuta@olemiss.edu

**Keywords:** electroencephalogram, episodic memory, synaptic plasticity

## Abstract

Sensory systems are widely known to exhibit adaptive mechanisms. Vision is no exception to input dependent changes in its sensitivity. Recent animal work demonstrates enhanced connectivity between neurons in the visual cortex. The purpose of the present experiment was to evaluate a human model that noninvasively alters the amplitude of the N1b component in the visual cortex of humans by means of rapid visual stimulation. Nineteen participants (M_age_ = 24 years; 52.6% male) completed a rapid visual stimulation paradigm involving black and white reversal checkerboards presented bilaterally in the visual field. EEG data was collected during the visual stimulation paradigm, which consisted of four main phases, a pre-tetanus block, photic stimulus, early post-tetanus, and late post-tetanus. The amplitude of the N1b component of the pre-tetanus, early post-tetanus and late post-tetanus visual evoked potentials were calculated. Change in N1b amplitude was calculated by subtracting pre-tetanus N1b amplitude from early and late post-tetanus. Results demonstrated a significant difference between pre-tetanus N1b (M = −0.498 µV, SD = 0.858) and early N1b (M = −1.011 µV, SD = 1.088), *t* (18) = 2.761, *p* = 0.039, d = 0.633. No difference was observed between pre-tetanus N1b and late N1b (*p* = 0.36). In conclusion, our findings suggest that it is possible to induce changes in the amplitude of the visually evoked potential N1b waveform in the visual cortex of humans non-invasively. Additional work is needed to corroborate that the potentiation of the N1b component observed in this study is due to similar mechanisms essential to prolonged strengthened neural connections exhibited in cognitive structures of the brain observed in prior animal research. If so, this will allow for the examination of strengthened neural connectivity and its interaction with multiple human sensory stimuli and behaviors.

## 1. Introduction

The visual cortex has been shown to exhibit input dependent adaptations. Specifically, the human visual cortex has been shown to adapt to color contrast [1], orientation [2], and patterns [3] in a selective fashion. However, it is not clearly understood whether these adaptations have to be specific to a particular aspect of visual input. It is not yet clearly known whether adaptations can happen to the input in general, in the absence of specificity.

Event-related potentials have been found to capture input dependent adaptations. Specifically, the amplitude of the N1 complex has been shown to be influenced by attention [4]. Moreover, the processing of sensory information in simple tasks (e.g., visual sensory stimuli) is further modulated by attention [4]. Altered processing of sensory information can impact the activity of other systems (e.g., memory systems) [5], therefore, the N1b component may be a marker indicating the effects of attention on memory. The N1b component is a late phase of the N1 complex that is a negative ERP signal occurring ~170–190-ms post stimulus onset [6,7]. Using similar paradigms to those employed by others [8,9], animal studies have observed similar results in the visual cortex of mice and rats [10,11,12,13].

In the present study, written as a brief report, our goal was to noninvasively examine the amplitude of the N1b component in the visual cortex of humans by multiple presentations of rapid visual stimulation. We hypothesized that bilaterally stimulating the human visual cortex with horizontal black and white reversal checkerboards would alter the N1b component of the visual cortex. If effective, this paradigm will provide an understanding of context-specificity of visual adaptation.

## 2. Materials and Methods

### 2.1. Subjects

A total of 19 right-handed college men and women participated in the present study. All subjects had normal or corrected vision. Informed consent was obtained from each participant. Due to the use of rapid visual stimulation, only participants who reported that they did not suffer from any neurological conditions, epilepsy, or migraine headaches were included. All procedures adhered to the rules of the Declaration of Helsinki and was approved by the University of Mississippi ethics committee (#19-047). All participants provided written consent prior to participation.

### 2.2. Apparatus

**Figure 1:**
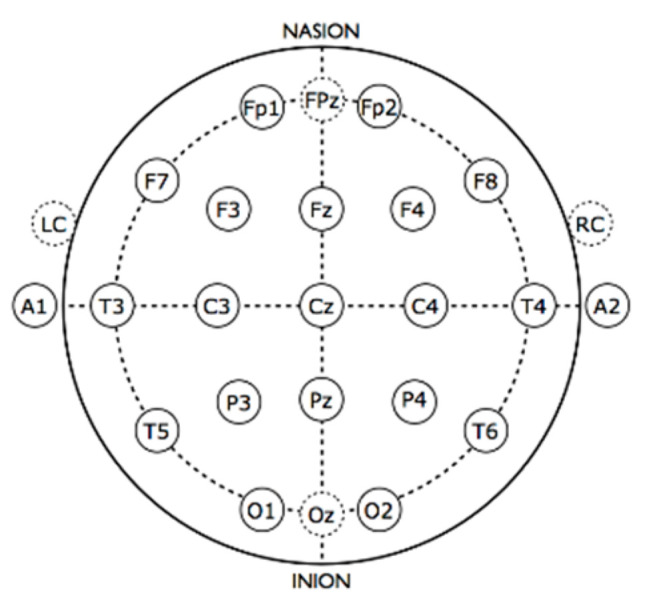
International 10–20 electrode placement.

EEG data were collected using a NuAmps 40 channel, 22-bit, digital amplifier (Compumedics Neuroscan, Charlotte, NC, USA), with a fitted 32-channel Quik-Cap with integrated bipolar leads for vertical and horizontal eye movement. Quik-Caps were manufactured of highly elastic breathable Lycra material with soft neoprene electrode gel reservoirs for enhanced patient comfort. All electrodes were placed according to the International 10–20 electrode placement standard (Figure 1). For the present study, electrodes of interest included T5, P3, Pz, P4, T6, O1, Oz, and O2. Electrodes embedded in the Quik-caps were made with sintered Ag/Ag/Cl electrodes because of their durability and ease of cleaning and re-use. The NuAmps system utilizes SCAN 4.3 software for data acquisition software and STIM2 (Compumedics Neuroscan) software to time-lock visual presentations to collect EEG data. EEG recordings were sampled at a continuous 2000 Hz (0.1–100 Hz bandpass filter). Electrode impedances were below 30 kΩ, which is considered an acceptable level for this system [14]. A common vertex reference (Cz) was used to acquire EEG, which was later referenced to the average off-line.

### 2.3. Stimuli

Visual stimuli consisted of black and white reversal checkerboards presented bilaterally in the visual field. The stimuli subtended 4° of visual angle from the vertical and horizontal midline. The visual angle was computed from the size and the distance of the image from the observer; visual angle calculation: A = (360/2π) (r/d) = 57.3(r/d), where A is the visual angle, pi(π) is approximately 3.14159, *r* is the size of the stimulus on the screen, and *d* is the distance of the observer from the screen. The stimuli were presented on a computer monitor (30-inch x flat-screen SVGA monitor with a resolution of 1920 × 1080 pixels Full HD at 60 Hz), 57 cm from subjects, and checkerboard luminance was at 100 percent contrast. Checkerboards were generated by custom software Stim2 (NeuroScan INC) on a Dell Pentium II/200 computer. TTL provided synchronization of stimulus events with EEG acquisition.

### 2.4. Procedure

**Figure 2:**
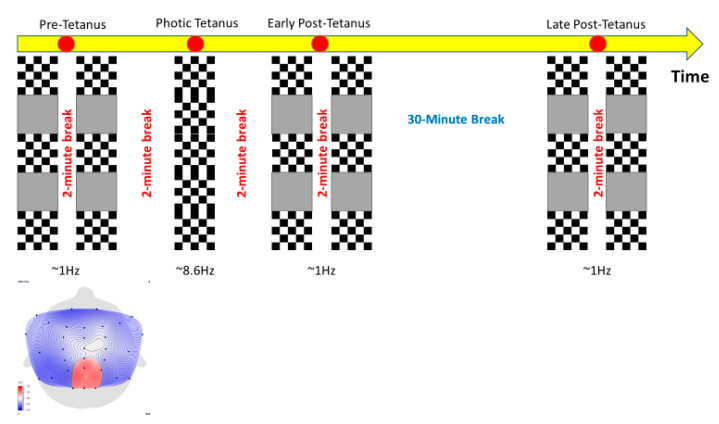
Visual stimulus paradigm. The pre-tetanus, early post-tetanus, and late post-tetanus blocks are comprised of interspersed presentations of 420 horizontal black and white reversal checkerboards presented for seventy seconds separated by fifteen seconds of a black screen with no checkerboards. During the pre-tetanus, early post-tetanus, and late post-tetanus blocks, the checkerboards were presented centrally at a rate of 1 Hz. The photic tetanus consisted of 1000 consecutive presentations of horizontal black and white reversal checkerboards presented centrally lasting for ~2-min. The pre-tetanus, early post-tetanus, late post-tetanus and photic tetanus blocks are separated by two-minute breaks (eyes closed) to allow for any retinal after-image to dissipate. A 30-min rest period occurred between the early post-tetanus and late post-tetanus.

See Figure 2 for a schematic of the procedure, which consisted of five phases: first, two pre-tetanus blocks; second, a photic stimulus; third, two early post-tetanus blocks; fourth, a 30-min rest period; and fifth, two late post-tetanus blocks. Each pair of pre-tetanus, early post-tetanus, and late post-tetanus was separated by a 2-min rest period with eyes closed. Moreover, after the photic tetanus, participants were instructed to close their eyes for two-minutes to allow any retinal after-image to dissipate. Participants, while resting their chin on a chin rest were required to fixate on a red circular dot in the center of the screen during data collection.

Each pre-tetanus, early post-tetanus, and late post-tetanus blocks lasted 10 min and comprised of interspersed presentations of 420 horizontal black and white reversal checkerboards presented for seventy seconds separated by fifteen seconds of a black screen with no checkerboards. During the pre-tetanus, early post-tetanus, and late post-tetanus blocks, the checkerboards were presented centrally at a rate of 1 Hz (stimulus-onset-asynchrony (SOA) range 950–1120-ms, duration 100-ms). A test frequency of 1 Hz was believed to be low enough not to have a physiological effect and not so long as to unnecessarily prolong the test blocks.

The photic tetanus consisted of 1000 consecutive presentations of horizontal black and white reversal checkerboards and lasted for ~2-min. Stimulus duration during the photic tetanus was 100-ms, with a randomly jittered inter-stimulus interval of 67–100-ms (temporal frequency: 8.6 Hz).

### 2.5. EEG Data Reduction

**Figure 3:**
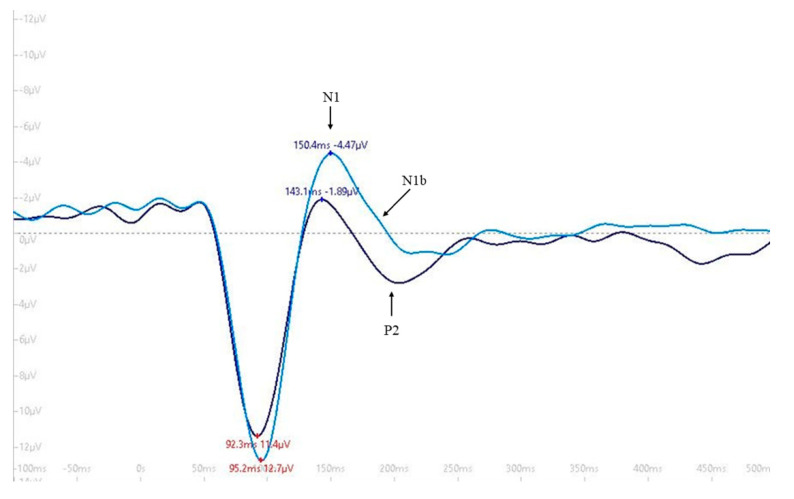
Visually evoked potential N1b waveform. Amplitude of the N1b component was defined as the mean amplitude of the section of the evoked potential, extending from the peak of the N1 component to halfway between the N1 and P2 components. N1b change was calculated by subtracting pre-tetanus N1b amplitude (dark blue line) from early (light blue line) and late post-tetanus.

EEG data for each participant was segmented with respect to event markers into 600-ms time epochs (100-ms before and 500-ms after stimulus onset) with epochs averaged by block (pre-tetanus, early post-tetanus, late post-tetanus). Epochs with eye blinks or artifacts were rejected and not included in the averaging process. The amplitude of the N1b component of the pre-tetanus, early post-tetanus, and late post-tetanus visual evoked potentials were averaged for clusters of electrodes (T5, P3, Pz, P4, T6, O1, Oz, and O2) positioned over the left and right posterior aspect of the skull and the occipital cortex, respectively (Figure 1). Clusters of electrodes were chosen based on capturing accurate and reliable data derived from prior studies [6,7]. In accordance with previously validated measures [6,8], the amplitude of the N1b component was defined as the mean amplitude of the section of the evoked potential, extending from the peak of the N1 component to halfway between the N1 and P2 components (see Figure 3 as an example). For each participant, we subtracted the pre-tetanus N1b amplitude from early and late post-tetanus.

### 2.6. Statistical Analysis

A Repeated Measures ANOVA (RM-ANOVA) was utilized to evaluate the changes in amplitude of the N1b component. Main effects for time (Baseline N1b, early N1b, and late N1b) were evaluated. Bonferroni-corrected paired t-tests were subsequently employed. Statistical significance was established as a nominal alpha of 0.05. All analyses were computed in JASP (v 0.11.1.0).

## 3. Results

Table 1 displays the characteristics of the sample. The sample, on average, was 24.05 ± 3.2 years, with 52.6% male.

**Figure 4:**
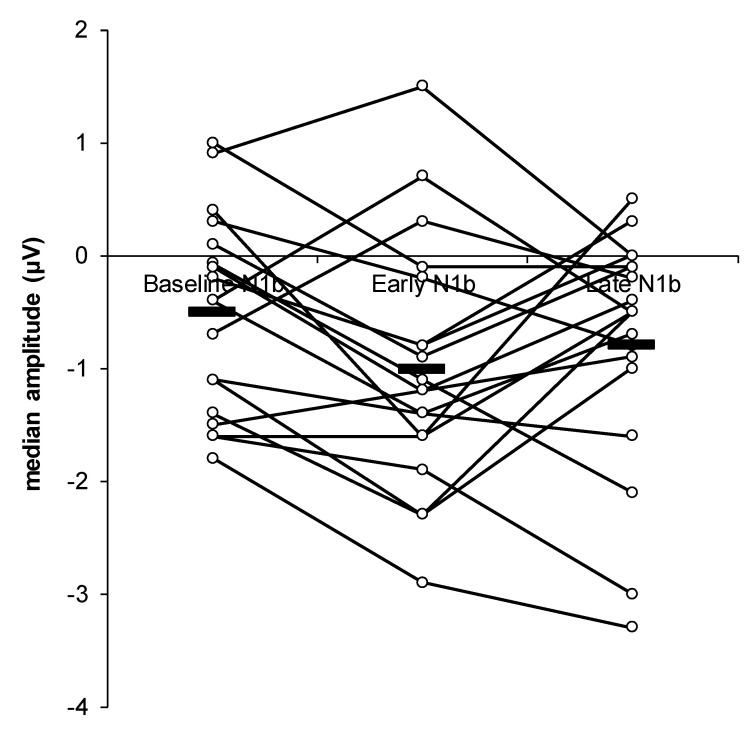
The change of the N1b component over time (baseline, early, and late) for individual participants. The “black line” shows the median amplitude at each time point.

**Figure 5:**
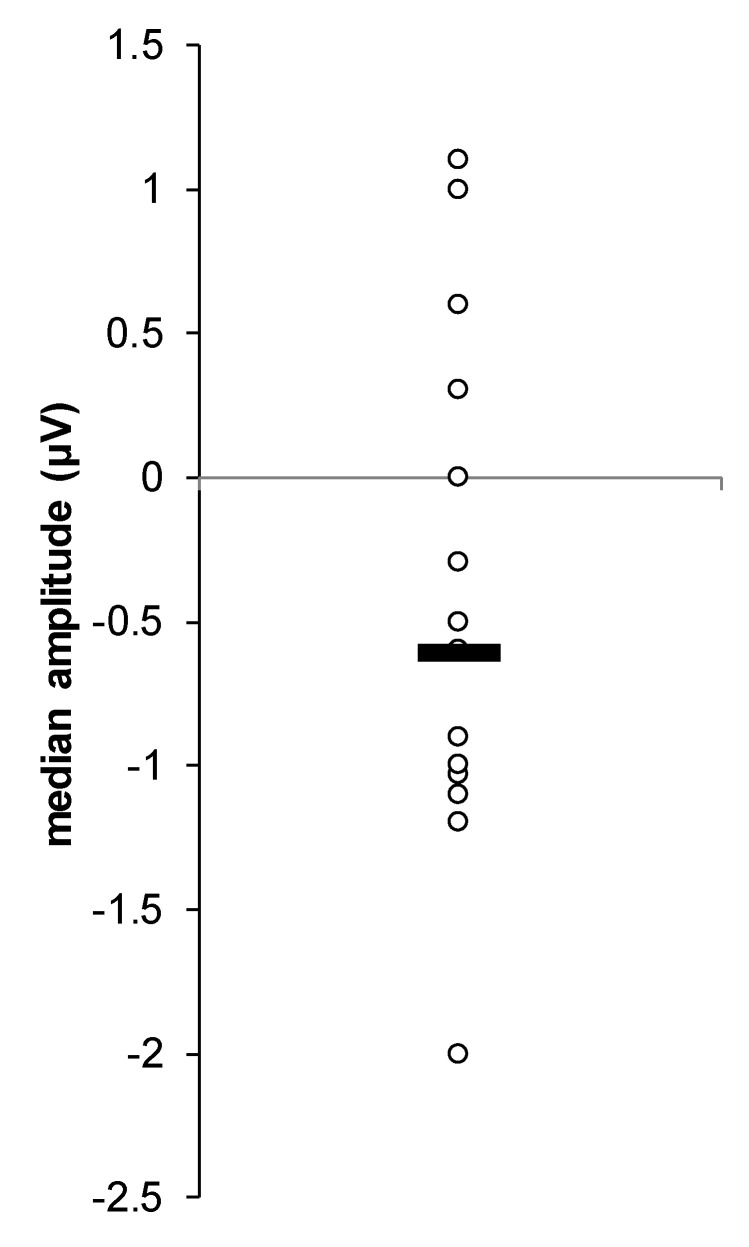
Individual differences between baseline and early-post tetanus. The “black line” shows the median difference.

Figure 4 displays the individual N1b results across the three time periods (baseline N1b, early N1b, and late N1b). The analysis of variance of the within-subjects’ effects indicated a non-significant main effect for time for N1b amplitude (F(2,36) = 2.99, *p* = 0.06). However, paired t-test post-hoc comparisons revealed a significant difference between baseline N1b (M = −0.498 µV, SD = 0.858) and early N1b (M = −1.011 µV, SD = 1.088), *t*(18) = 2.761, *p* = 0.039, d = 0.633. See Figure 5 for individual level differences between baseline N1b and early N1b. There was no significant difference between baseline N1b (M = −0.498 µV, SD = 0.858) and late N1b (M = −0.784 µV, SD = 1.039), *t*(18) = −0.936, *p* = 0.362, d = −0.215.

## 4. Discussion

The present study replicates that rapid visual stimulation produces changes in the amplitude of the visual evoked potential N1b waveform in the visual cortex measured by EEG, as previously shown [8]. After the photic tetanus, a significant increase in the N1b component was observed in the early post-tetanus bilaterally but not in the late post-tetanus.

These results support the findings of Teyler et al. [8] that visually evoked cortical responses recorded non-invasively [13] in humans can be potentiated after rapid visual stimuli. Teyler et al. [8] demonstrated that, among a sample of 6 right-handed males, presenting checkerboards to the left or right visual field resulted in a significant post-tetanus N1b component in the hemisphere contralateral to the tetanus. However, the potentiation of the N1b component declined in the subsequent blocks [8]. The decline in the N1b component may be a reflection of low-frequency stimulation, therefore, causing depotentiation [15]. Likewise, Ross et al. [7] also experienced a significant increase in the N1b component early-post tetanus after exposing participants to horizontal and vertical sine gratings. Similar to Teyler et al. [8], they also observed a decline in the potentiation of the visual evoked potential in later blocks [6]. Our results align with these findings, in that, on average, early N1b was lower than baseline N1b. Yet, some participants produced higher early N1b values than baseline values. Differences in responses to the visual stimuli between time points and individuals may, in part, be due to varying levels of attention. Nevertheless, our paradigm suggests that neurons in the primary visual cortex are responsive to a unidirectional stimulus. In short, the decoding of visual sensory information leading to visual perception by the brain is determined by neural coding related to orientation, motion, and color [16,17]. Mutually, these features are incorporated by the visual system to organize pieces of sensory information for object recognition similar to orienting pieces of a puzzle. Prior research suggests that neurons in the visual cortex may be orientation specific [17]. However, our study incorporated a horizontal only visual stimulus that produced significant changes in the neural activity of the visual cortex. The visual paradigm incorporated in this study differed from previous studies in that they used a combination of circular horizontal and vertical sine gratings [7] and presented the visual stimuli to the left or right visual field [8]. Collectively, these findings may suggest that the visually-evoked change in the amplitude of the N1b component may be mediated by attention or synaptic potentiation in the visual cortex, functioning similar to persistent strengthening of synapses in cognitive structures of the brain, such as the hippocampus. The visual cortex plays an important role in processing and organizing visual information, ultimately assisting in spatial-learning and delayed visual recognition memory [5]. Nevertheless, future studies should focus on the mechanisms driving these findings. Although our study design is similar to other related work [8,18], future work would benefit by including a control condition.

In summary, our replication findings indicate that it is possible to induce changes in the N1b visually evoked potential in the visual cortex of humans non-invasively. We demonstrate this effect using a large, mixed-gender sample. Additional work is needed to corroborate that the potentiation of the N1b component observed in this study is due to similar mechanisms essential to the persistent strengthening of synaptic potentiation in the cognitive structures of the brain observed in prior animal research. If so, this will allow for the examination of increased neural strength and connectivity and its interaction with multiple human sensory stimuli and behaviors.

## Figures and Tables

**Table 1:** Characteristics of the sample (N = 19).

Variable	Point Estimate	SD
Age, mean years	24.05	3.2
Gender, % Male	52.6	
BMI, mean kg/m^2^	25.28	4.81

BMI, Body Mass Index; SD, Standard Deviation.
